# Supplementary material for: Complexome profiling of the Chlamydomonas psb28 mutant reveals TEF5 as an early PSII assembly factor
Source: Plant Cell. 2025 Mar 17;37(6):koaf055. doi: 10.1093/plcell/koaf055 (PMC12164590; doi:10.1093/plcell/koaf055)
Supplement: koaf055_Supplementary_Data [file koaf055_supplementary_data.zip › psb28_interactive.zip!/plots/intensity/qualityPlots/peptides/_Cre03.g154600.t1.2.html]

### Cre03.g154600.t1.2

Trivial name:   
SeqCoverage [median]: 14.60  
Mass: 34.56 kDa  
  
 

| Sequence | MissedCleavage | Mass [Da] | wtIntensity | muIntensity | Score |
| --- | --- | --- | --- | --- | --- |
| APPVKDGQQ | 1 | 938.48 | 347.39 | 0.00 | 87.32 |
| DKLNSELSEFSQR | 1 | 1551.75 | 3052.48 | 18895.13 | 134.68 |
| FGNLFYVR | 0 | 1014.53 | 1658.88 | 2743.44 | 99.92 |
| IYTAYGPGTTPPEADLR | 0 | 1820.89 | 4311.90 | 12712.15 | 245.69 |
|  |  |  | 9370.6 | 34350.7 |  |
